# Metabolic effects of milk protein intake strongly depend on pre-existing metabolic and exercise status

**DOI:** 10.1186/1743-7075-10-60

**Published:** 2013-10-02

**Authors:** Bodo C Melnik, Gerd Schmitz, Swen Malte John, Pedro Carrera-Bastos, Staffan Lindeberg, Loren Cordain

**Affiliations:** 1 Department of Dermatology, Environmental Medicine and Health Theory, University of Osnabrück, Osnabrück, Germany; 2 Institute of Clinical Chemistry and Laboratory Medicine, University Clinics of Regensburg, Regensburg, Germany; 3 Center for Primary Health Care Research, Lund University, Lund, Sweden; 4 Department of Health and Exercise Science, Colorado State University, Fort Collins CO 80523, USA

**Keywords:** Adipogenesis, Body weight, Branched-chain amino acids, Glutaminolysis, Insulin resistance, Milk proteins, microRNA, mTORC1, Obesity, Prostate cancer

## Abstract

Milk protein intake has recently been suggested to improve metabolic health. This Perspective provides evidence that metabolic effects of milk protein intake have to be regarded in the context of the individual’s pre-existing metabolic and exercise status. Milk proteins provide abundant branched-chain amino acids (BCAAs) and glutamine. Plasma BCAAs and glutamine are increased in obesity and insulin resistance, but decrease after gastric bypass surgery resulting in weight loss and improved insulin sensitivity. Milk protein consumption results in postprandial hyperinsulinemia in obese subjects, increases body weight of overweight adolescents and may thus deteriorate pre-existing metabolic disturbances of obese, insulin resistant individuals.

## Introduction

Recently McGregor and Poppitt suggested milk protein intake for the improvement of metabolic health [1]. However, the authors missed important insights into branched-chain amino acid (BCAA) metabolism under conditions of obesity and insulin resistance. They emphasized beneficial effects of milk protein ingestion for skeletal muscle but ignored adverse effects of BCAAs on adipose tissue and long-term β-cell homeostasis. Obviously, milk’s physiological function promoting neonatal growth is not restricted to the musculoskeletal system. It is the intention of this Perspective article to demonstrate that the evaluation of metabolic effects of milk protein consumption has to consider the nutritional and endocrine status and the level of physical activity of the milk protein consumer.

### Milk proteins increase BCAA influx

**Figure 1:**
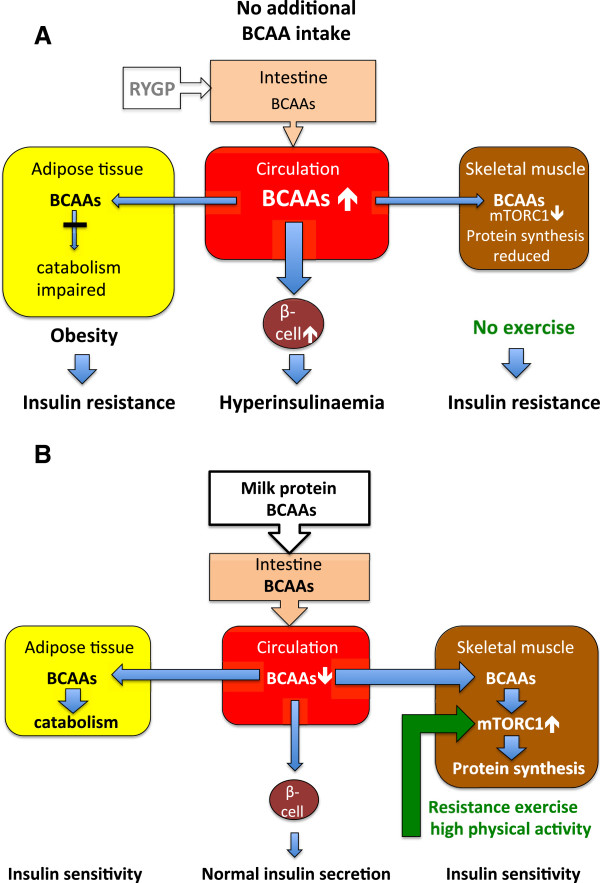
**BCAA metabolism in obese, sedentary subjects versus healthy, physically active individuals. A**. Deviated BCAA metabolism in obese, sedentary individuals. **B**. BCAA metabolism in healthy physically active individuals. BCAA = branched-chain amino acids (leucine, isoleucine, valine). RYGP = Roux-en-Y gastric bypass surgery.

Plasma BCAAs (leucine, isoleucine, valine) and glutamine/glutamate are increased in obesity, insulin resistance and type 2-diabetes (T2D) [2-9]. An extra daily intake of 53 g milk protein but not 53 g meat increased serum insulin and insulin resistance in 8-year-old boys [10]. Impaired BCAA catabolism of adipocytes is a crucial metabolic deviation of obesity [11,12] (Figure 1A). As BCAA plasma levels in obesity are already elevated an additional BCAA influx may further deteriorate the pre-existing metabolic imbalance. In fact, the marked decrease in BCAA plasma levels resulting from gastric bypass surgery is associated with weight loss and improved insulin sensitivity [13,14]. Palaeolithic, physically active hunter-gatherers consumed *structural proteins* like fish and meat. In contrast, modern Neolithic humans have “mutated” into physically inactive individuals, who particularly consume *signalling proteins from milk* providing abundant “fast dietary proteins” leading to high plasma BCAA and glutamine levels [15]. Palaeolithic dairy-free diets exhibit lower insulin levels with improved insulin sensitivity protecting against the development of diseases of civilization [16-19].

### Mechanical stimuli increase mTORC1-mediated muscle protein synthesis

According to our opinion it may be hazardous to promote milk protein consumption especially in obese individuals with sedentary lifestyles and/or insulin resistance. Although BCAAs are required for mTORC1-dependent muscle protein synthesis [20,21], BCAA intake alone is not sufficient to significantly increase muscle mass. Mechanical stimuli, which activate mTORC1 in myocytes, are of critical importance to cause significant skeletal muscle hypertrophy [22,23] (Figure 1B). Intriguingly, eccentric muscle contractions increase mTORC1 activation by phosphorylation of tuberous sclerosis complex-2 (TSC2) associated with the translocation of TSC2 away from the lysosome [24]. According to a recent model, mTORC1 activation occurs at the lysosome and is mediated through an amino acid-sensing cascade involving RAG GTPases, Ragulator and vacuolar H^+^-ATPase [25,26]. Whey proteins contain highest amounts of BCAAs [27-29]. Whey protein intake after resistance exercise substantially increased mTORC1 activity in skeletal muscle of healthy young men [30]. Thus, weight loss and muscle gain in conjunction with increased milk protein intake may only be successful in combination with enhanced physical activity (Figure 1B) [31]. In contrast, additional intake of BCAAs in sedentary obese subjects may further deteriorate metabolic control resulting in hyperinsulinemia, insulin resistance and T2D (Figure 1A).

### Milk protein consumption and postprandial hyperinsulinemia in obesity

Of all animal proteins, whey proteins contain the highest amount of leucine (14% of total amino acids) [27]. Whey proteins are “fast proteins” [15], that are hydrolysed within minutes in the intestine and immediately increase BCAA levels in the systemic circulation thus functioning like an intravenous administration of amino acids [29,32]. In healthy lean individuals oral administration of leucine induces postprandial hyperinsulinemia especially in combination with increased glucose intake [33,34]. In obese subjects, oral administration of leucine induces exaggerated and prolonged postprandial hyperinsulinemia without significant changes of blood glucose levels [34-36]. In children, daily intake of 53 g milk protein, but not of 53 g meat induced hyperinsulinemia and insulin resistance under fasting conditions [10]. In overweight adolescents daily intake of 35 g whey protein or casein significantly increased fasting plasma insulin levels [37]. Thus, there is substantial evidence that increased milk protein consumption in obese individuals persistently over-stimulates insulin secretion, which in the long term may promote early onset of β-cell apoptosis. Milk-stimulated islet cell hyperplasia and β-cell hyperresponsiveness are obviously physiological metabolic effects that promote anabolism and postnatal growth. However, experimental evidence in β-cell TSC2^−/−^ deficient mice indicates that persistently increased mTORC1-mediated β-cell stimulation from the beginning of postnatal life induced hyperinsulinemia and enhanced β-cell proliferation during adolescence but early onset of β-cell apoptosis with decreased insulin secretion in adulthood [38].

### Milk consumption, obesity and risk of type 2-diabetes

Milk ingestion is a novel human behaviour introduced by the Neolithic revolution, industrially maximized by widespread refrigeration technology [39]. The *NHANES*[40] and the *Growing-Up Today Study*[41] observed increased BMI in children and adolescents in association with increased milk consumption. Weight gain associated with increased milk intake has also been observed in healthy adults [42]. Supplementation of milk protein (either 35 g whey protein, skim milk protein, or casein) to overweight adolescents further increased body weight [37]. Accordingly, chronic leucine supplementation in rats on a high-fat diet further increased body weight [43]. Leucine plays a pivotal role for mTORC1 activation [44-46] including adipocytes [47,48]. Leucyl-tRNA synthetase is another recently identified key mediator for BCAA-induced mTORC1 activation [49]. mTORC1 is a central regulator of adipogenesis linking BCAA abundance to mTORC1-driven obesity [50]. BMI is a critical determinant for the induction of pubertal growth. Notably, the *NHANES* study found a correlation between milk consumption in children with increased BMI [40] and early age of menarche [51], a risk factor for the development of obesity, T2D and metabolic syndrome [52-56]. Milk proteins are enriched in glutamine [57], the precursor of the glutaminolysis pathway, which plays a crucial role for insulin secretion [58]. Remarkably, glutamate dehydrogenase is allosterically activated by leucine [59,60]. Thus, leucine- and glutamine overload by high milk protein consumption, may permanently over-stimulate insulin secretion and mTORC1 signalling. In fact, elevated plasma levels of BCAAs and glutamate are positively correlated with increased BMI and insulin resistance [4,7].

### Increased BCAA availability and insulin resistance

There is compelling evidence that amino acid availability regulates S6 kinase and multiple translation factors [61]. BCAAs by increasing mTORC1-S6K1 signalling act as positive signals for maintenance of protein stores, while inhibiting other actions of insulin at multiple levels [62]. In amino acid-infused humans, over-activation of mTORC1-S6K1 pathway increased inhibitory insulin receptor substrate (IRS)-1 phosphorylation at Ser312, Ser636/639 and Ser1101 resulting in insulin resistance of skeletal muscle [63-65]. Thus, there is substantial evidence that inappropriate activation of mTORC1-S6K1 signalling by amino acids induces insulin resistance, the fundamental metabolic deviation leading to T2D [9,63-66]. Whey proteins in contrast to meat proteins provide fast hydrolysable BCAAs comparable to a BCAA infusion promoting insulin secretion and insulin resistance, major intrinsic mechanisms of milk signalling [10,67].

### Milk protein consumption and risk of prostate cancer

Nutrition plays an important role in mTORC1-driven cancer development [26,46,68,69]. mTORC1 steers prostate cancer (PCa) initiation and metastasis [69]. Accumulating evidence links PCa initiation and progression to increased milk protein consumption and milk-mediated activation of mTORC1 [70]. *The European Prospective Investigation into Cancer and Nutrition* confirmed that high intake of dairy protein is associated with an increased risk of PCa [71]. A 35 g/day increase in dairy protein intake was associated with an increased risk of PCa of 32% [71]. Furthermore, increased PCa-specific mortality has recently been associated with increased whole milk intake [72]. In contrast to meat, milk and milk protein fractions contain substantial amounts of exosomal microRNAs, predominantly microRNA-21 [73-75], that is an oncogenic and adipogenic microRNA [76,77]. Remarkably, addition of commercial milk to PCa cell cultures increased the proliferation of cancer cells by 30% [78]. Furthermore, commercial milk contains substantial amounts of the let-7 microRNA family [75]. Notably, it has recently been demonstrated that over-expression of let-7 induced insulin resistance [79,80].

## Conclusions

There is no evidence that milk proteins *per se* improve metabolic health. In contrast, increased consumption of milk proteins may further impair BCAA metabolism of obese, insulin resistant, sedentary individuals. It is now clear that not calorie restriction but BCAA restriction extends lifespan in *Drosophila melanogaster*[81,82]. Reduction of BCAA intake with reduced mTORC1 activation explains the metabolic benefits of dietary restriction [83,84]. Persistent leucine-mediated hyperinsulinemia in obesity induced by persistent milk protein consumption may promote an earlier onset of β-cell apoptosis. Epidemiological evidence underlines the association between increased milk intake and higher BMI, increased milk intake and early onset of menarche, and the association of increased BMI as well as early menarche and increased risk of T2D. Thus, we recommend a more careful and restricted use of milk proteins, especially in the setting of pre-existent obesity, insulin resistance as well as sedentary life style.

## Abbreviations

AA: Amino acids; BCAA: Branched-chain amino acid; GDH: Glutamate dehydrogenase; BMI: Body mass index; microRNA: micro-ribonucleic acid; mTORC1: mechanistic (mammalian) target of rapamycin complex 1; PCa: Prostate cancer; RYGS: Roux-en-Y-gastric bypass surgery; T2D: Type 2-diabetes.

## Competing interests

BCM, GS, SMJ, PCB, SL and LC have declared no competing interests.

## Authors’ contributions

BCM wrote the manuscript. BCM, GS, SMJ, PCB, SL and LC searched and critically reviewed the literature. All authors read and approved the final manuscript.
